# Comparison of two methods of locating proximal femoral nail anti-rotation in the treatment of femoral intertrochanteric fractures

**DOI:** 10.1186/s13018-020-01614-9

**Published:** 2020-03-17

**Authors:** Rong-hua Tian, Qin-ming Zhang, Feng-long Chu, Xiao-yan Li, Zhen Jiang, Liang Han, Peng Sun, Hai-bin Wang, Yu-lei Chi, Bin Wu

**Affiliations:** 1 Department of Clinical Medicine, Jining Medical University, No. 133, Hehua Road, Taibai Lake New District, Jining City, 272067 Shandong Province China; 2 Department of Orthopedic, Affiliated Hospital of Jining Medical University, No. 89, Guhuai Road, Jining City, 272029 Shandong Province China

**Keywords:** Three-point positioning method, Common positioning method, Intertrochanteric fractures of femur, Helical blade, Proximal femoral nail anti-rotation (PFNA)

## Abstract

**Background:**

To compare the efficacy of three-point locating versus routine locating techniques for implanting helical blades for proximal femoral nail anti-rotation-II in the treatment of trochanteric fractures.

**Methods:**

From January 2010 to June 2013, 90 patients with intertrochanteric fractures were surgically treated, including 48 males and 42 females with an average age of 70.5 ± 7.2 years. According to the AO classification, there were 45 cases of A2.1, 35 cases of A2.2, and 10 cases of A2.3. Based on locating techniques, the 90 patients were divided into two groups: the three-point group and the routine group, with 45 patients in each group. All operations were performed by the same group of surgeons using proximal femoral nail anti-rotation (PFNA); the helical blade was inserted into the femoral neck with the three-point locating technique or by the usual method according to treatment group. Several figures including total operation time, elapsed time for implanting the helical blade, intraoperative blood loss, X-ray exposure time, and tip-apex distance (TAD) were measured and compared.

**Results:**

The three-point group was significantly superior as compared to the routine group in terms of total operation time [(59.34 ± 9.42) min vs (67.61 ± 12.63) min, *P* < 0.01], elapsed time for implanting the helical blade [(4.58 ± 1.25) min vs (7.82 ± 2.19) min, *P* < 0.01], intraoperative blood loss [(92.78 ± 34.09) ml vs (154.01 ± 39.10) ml, *P* < 0.01], X-ray exposure time [(8.84 ± 1.45) vs (14.62 ± 2.91), *P* < 0.01], and tip-apex distance [(16.78 ± 1.55) mm vs (21.91 ± 3.01) mm, *P* < 0.01]. Among the 90 patients, 80 were followed up for an average time of 12 months (10–15 months), including 42 patients who were part of three-point group and 38 patients who were part of the routine group. No spiral blade cut was found on the femoral head in any patient in the three-point group, whereas it occurred in 2 patients in the routine group 1 month after surgery. However, there was no significant difference in the Harris score between the two groups 6 months after the operation.

**Conclusion:**

The three-point locating method is faster and more accurate than the routine locating method.

## Background

Femoral intertrochanteric fracture is one of the most common senile fractures [1], accounting for 3.1% of adult fractures, and the incidence is increasing every year. It is one of the most important causes of mortality in the geriatric population [2]. At present, most tend to be treated with intramedullary fixation [3]. In the past, when inserting the femoral neck spiral blade, the visual method was often used to locate the screw blade, and the ideal position was finally obtained by adjusting it many times; some cases could not obtain the ideal position because of the difficulty of nail path diversion. The trauma was large, the operation time was prolonged, and the amount of bleeding was also profuse. In recent years, some scholars have attempted to insert spiral blades under X-ray fluoroscopic navigation and achieved satisfactory results [4–6]. However, due to the limitations of instruments and technology, it could only be carried out in a few major hospitals. Since January 2010, our department has gradually explored a new three-point positioning method to insert the femoral neck spiral blade. Once successfully implanted, the number and length of X-ray exposures were significantly reduced, and the accuracy was improved. The reports are as follows.

## Methods

### General data

From January 2010 to June 2013, 90 cases of intertrochanteric fracture of the femur were treated surgically. There were 48 males and 42 females aged from 62 to 85 years (mean 70.5 years). The causes of injury were car accidents in 11 cases and falls in 79 cases; the AO classification was A2.1 in 45 cases. There were 35 cases of type A2.2 and 10 cases of type A2.3, all of which were closed fractures. Internal fixation was performed 4 to 9 days after admission. The exclusion criteria were pathological fractures and patients with severe medical diseases and multiple injuries. According to the length of hospitalization, 45 patients in each group were treated with PFNA internal fixation and operated by the same group of doctors. The three-point positioning method was used to insert the spiral blade in the three-point group, and the ordinary fluoroscopic positioning method was used in the routine group.

### Surgical methods

**Fig. 1:**
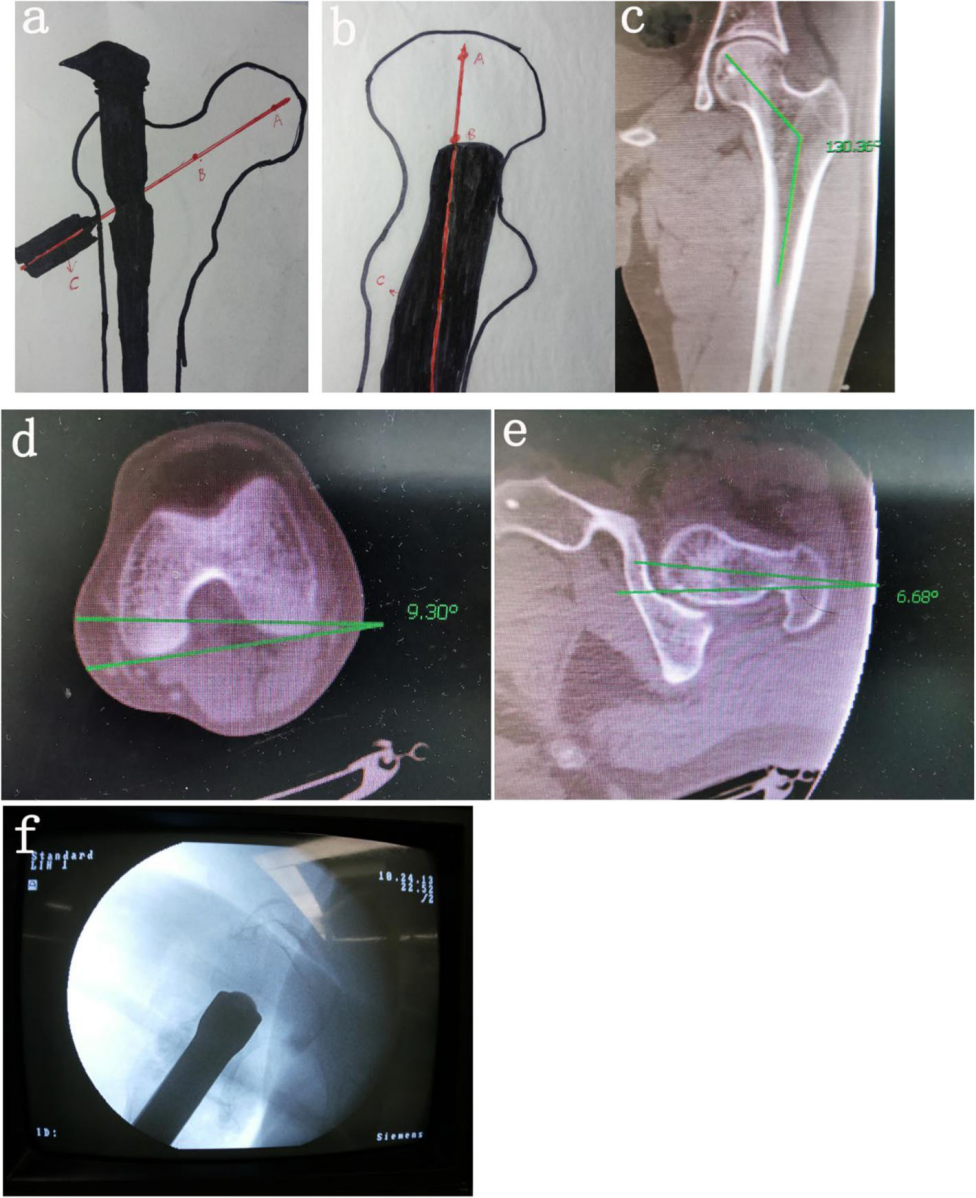
Schematic diagram of three-point positioning. **a** Intraoperative positive fluoroscopy, in which points A, B, and C are on or near a straight line. **b** During the operation, using lateral fluoroscopy positioning, the three points A, B, and C were in-line with the guiding needle. **c** The neck shaft angle was measured. **d** The angle between the posterior connecting line of the femoral condyle and the horizontal line was measured. **e** The angle between the femoral neck and the horizontal plane was measured. **f** In the lateral position, the A, B, and C are on a line

In each patient, the full-length CT scan of the contralateral femur was performed before operation, and femoral collo-diaphyseal angle was measured from the coronal plane of CT (Fig. 1c). The angle between the posterior connecting line of the femoral condyle and the horizontal line was measured at the distal end of the femur, and the angle between the femoral neck and the horizontal plane was measured at the proximal end of the femur. The difference between these two angles is the femoral neck anteversion (FNA), which is 9.3 + 6.68 = 15.98° (Fig. 1d, e).

**Fig. 2:**
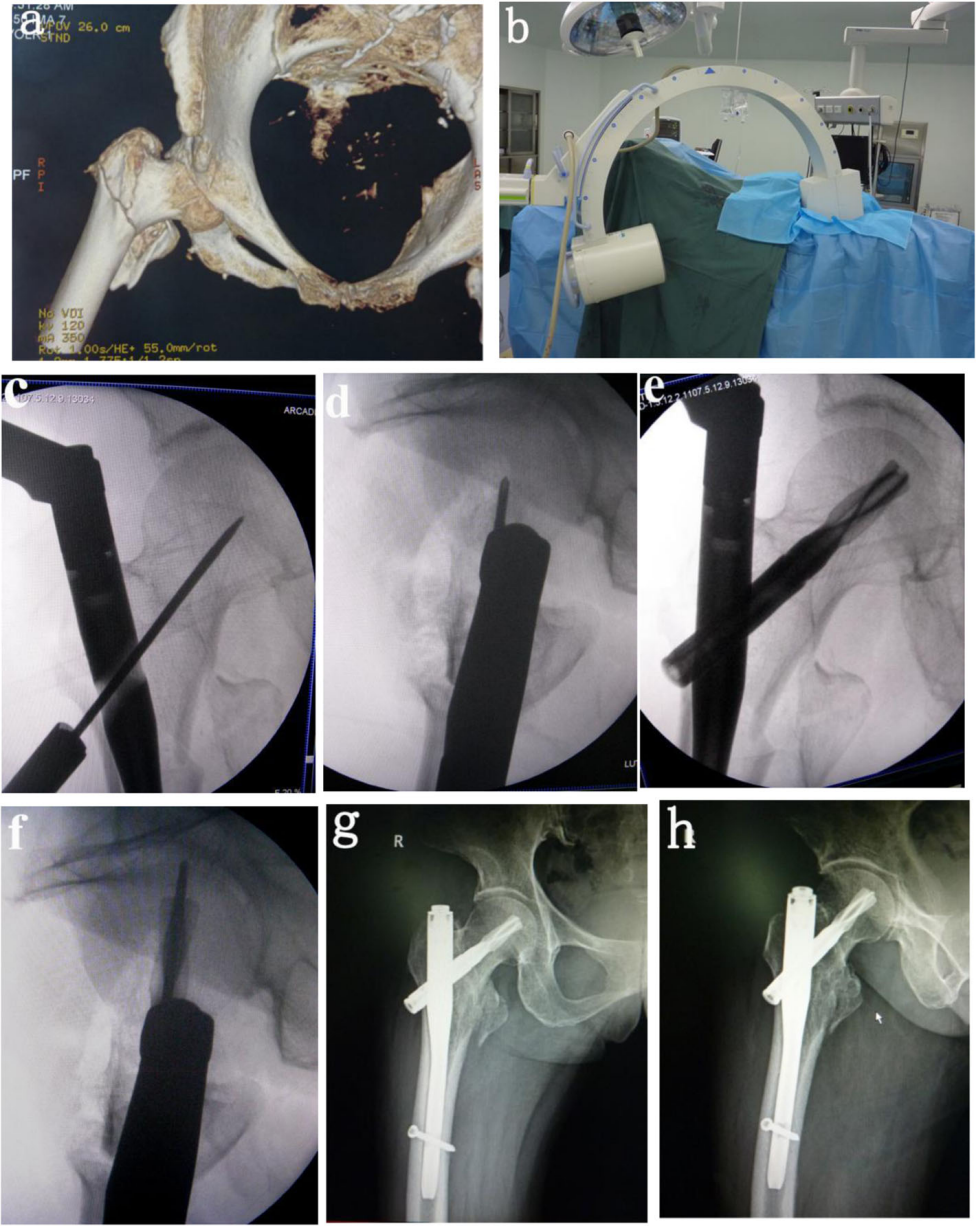
Female, 72 years old, right femoral intertrochanteric fracture caused by road traffic injury (AO A2.1). Surgical treatment was performed 3 days after admission. A three-point positioning method was used to implant femoral neck screws. **a** Preoperative three-dimensional CT examination showed a right intertrochanteric fracture and a lesser trochanter fracture with displacement. **b** During the operation, the fluoroscope was tilted 15° to show the lateral image of the femoral neck. **c** Intraoperative anteroposterior fluoroscopy 3-point positioning to ensure that the guiding needle was in the middle of the femoral neck. **d** During the operation, using lateral fluoroscopy, the 3 points were located to ensure that the guiding needle was in the middle of the femoral neck**. e** Perspective positive position after placing the spiral blade. **f** Perspective on the posterior lateral position when inserting the spiral blade. **g**, **h** The X-rays were followed up 2 months after surgery

**Fig. 3:**
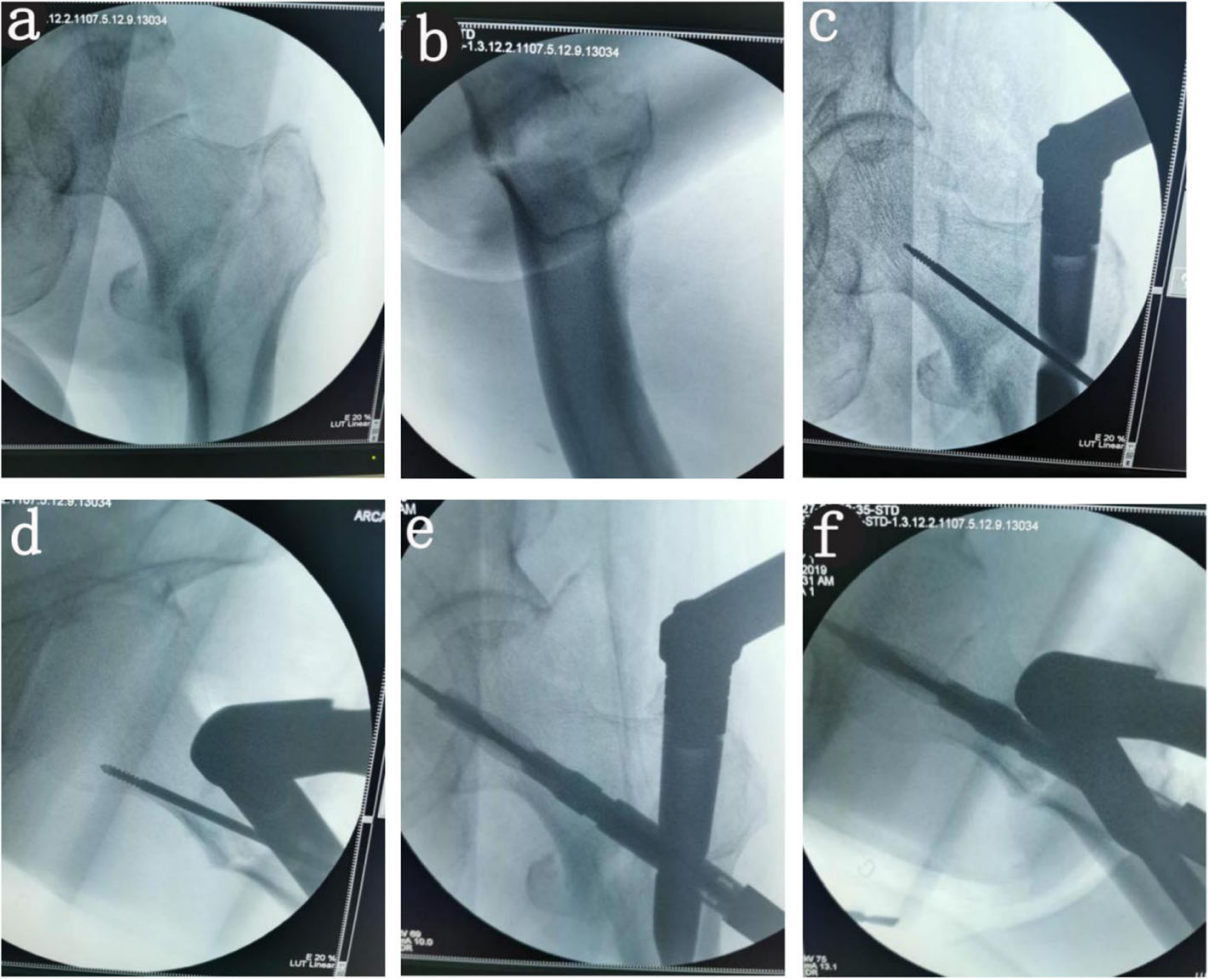
Female, 87 years old, left femoral intertrochanteric fracture caused by fall at home (AO A2.1). Surgical treatment was performed 5 days after admission. The femoral neck screw was implanted by routine locating techniques. **a** Preoperative X-ray examination (AP posture). **b** Preoperative X-ray examination (lateral position). **c** Intraoperative anteroposterior fluoroscopy shows that the guide needle was in the middle of the femoral neck. **d** Intraoperative lateral fluoroscopy shows that the guiding needle was in the anterior of the femoral neck. **e** Perspective anteroposterior position after placing the spiral blade. **f** Perspective on the posterior lateral position when inserting the spiral blade shows the blade in the anterior of the femoral neck

Patients with severe cardiopulmonary disease who had difficulty in extubation were performed with epidural anesthesia, and other patients were performed with intravenous inhalation combined anesthesia; the patient was laid on supine, and an orthopedic traction bed was placed. First, traction reduction was performed. When fluoroscopy showed that the fracture reduction was satisfactory, the patient was disinfected, laid out, and an incision of approximately 4 cm was made on the vertex of the proximal greater trochanter to expose the apex of the greater trochanter. Under fluoroscopy, the vertex of the greater trochanter was selected as the nail entry point; the proximal opening was opened, and the guide needle was inserted; the 180~200-mm nail was selected according to the fracture type. The three-point group used the three-point positioning method. First, the sleeve was installed, and the depth of the main nail was adjusted. When the axis of the sleeve was in place, the center of the femoral neck and the center of the femoral head (Figs. 1 and 2c) were on or near a straight line, and then the imaging approach was changed to lateral fluoroscopy. The angle between the perspective angle and the horizontal plane was approximately 15° (by we measured before operation) (Fig. 2b). According to the degree of rotation of the patient’s lower extremities, the lateral image of the femoral neck was viewed. When the axis of the sleeve was in place, the center of the femoral neck and the center of the femoral head formed a line (Figs. 1b and 2d). The needle was inserted directly into the guiding needle; the depth of the nail was approximately 5 mm; the extended needle was inserted into the spiral blade (Fig. 2e, f). In the conventional group, the insertion depth and forward inclination angle of the main nail were visually measured by the ordinary positioning method; the guiding needle was inserted after satisfactory adjustment, and the spiral blade was inserted along the guide needle (Fig. 3a–f).

For example, the neck shaft angle which we measured in the three-point group was 130° (Fig. 1c) because the angle of the PFNA screw blade and the main nail are also 130°; it must pass through the midpoint of the femoral head (point A) as long as the guiding needle passes through the midpoint of the femoral neck (point B) under correct reduction, and their outward extension line is point C. In the lateral position, in the same way, we have measured the anteversion angle; after reduction, we put the patella forward, and then choose the measured anteversion angle as the tilt angle of the C-arm, which must be a line in the lateral position. If the guide needle is inserted directly at this time, it must pass through the midpoint of the lateral position of the femoral neck (point B) and the midpoint of the lateral position of the femoral head (point A). Point C is determined when we insert the guide needle; it is located at the midpoint below the greater rotor, and this point is determined as long as the opening position is correct. If point B or point A is not in the central position, there must be a problem with our reduction, and the anteversion of the femoral neck needs to be adjusted. Then, the rest can be done according to the usual method.

### Postoperative management

Postoperative aseptic dressing was used with routine analgesic treatment for 3 days, and the dressing was replaced 24 h after the operation. Antibiotics were discontinued within 24 h after the operation, and functional exercises with an ankle pump on the affected limb under the guidance of rehabilitation specialists were scheduled on the second day after operation. Functional exercise of the hip and knee joint was performed 48 h after the operation, and walking without weight bearing was performed with the assistance of a walking aid. The stitches were removed 2 weeks after the operation, and weight bearing walking began 8 weeks after the operation.

### Evaluation indicators and statistical analysis

The total operation time, the implantation time of the spiral blade, the amount of blood loss during the operation, and the exposure time of the spiral blade were recorded. The distance between the tip of the spiral blade and the cortical bone of the femoral head was measured on the postoperative X-ray film, and the sum of the distance between the positive image and the lateral image was the apex distance. The data were analyzed by the SPSS 19 statistical software (IBM, Armonk, USA). All the data were expressed as mean values, and there was a significant difference between the two groups by *t* test (*P* < 0.05).

## Results

### Early results

The operation time, the time of placing the spiral blade, the amount of blood loss, the exposure time, and the tip distance of the two groups are shown in Table 1. The three-point group was superior to the routine group in five indexes, and the differences of each index between the two groups were statistically significant (Table 2). Typical cases are shown in Fig. 2.

**Table 1:** Comparison of the main characteristics of the patients included in the study

Indicator	Three-point method group, group A; *n* = 45	Conventional method group, group B; *n* = 45	*P*
Sex: male/female	28/17	23/22	0.52
Age (years): mean (range)	72.02 ± 6.26	70.05 ± 5.77	0.55
Side: right/left	20/25	21/24	0.93
Mechanism of injury			
Fall at home	39	36	0.45
Traffic accident	6	9	
Associated comorbidities			
Hypertension	28	30	0.87
Diabetes	12	13	
Cardiovascular disease	8	9	
Neurological disease	6	7	
AO fracture classification (Fig. 4)			
A2.1	23	22	0.68
A2.2	20	15	
A2.3	6	4

**Fig. 4:**
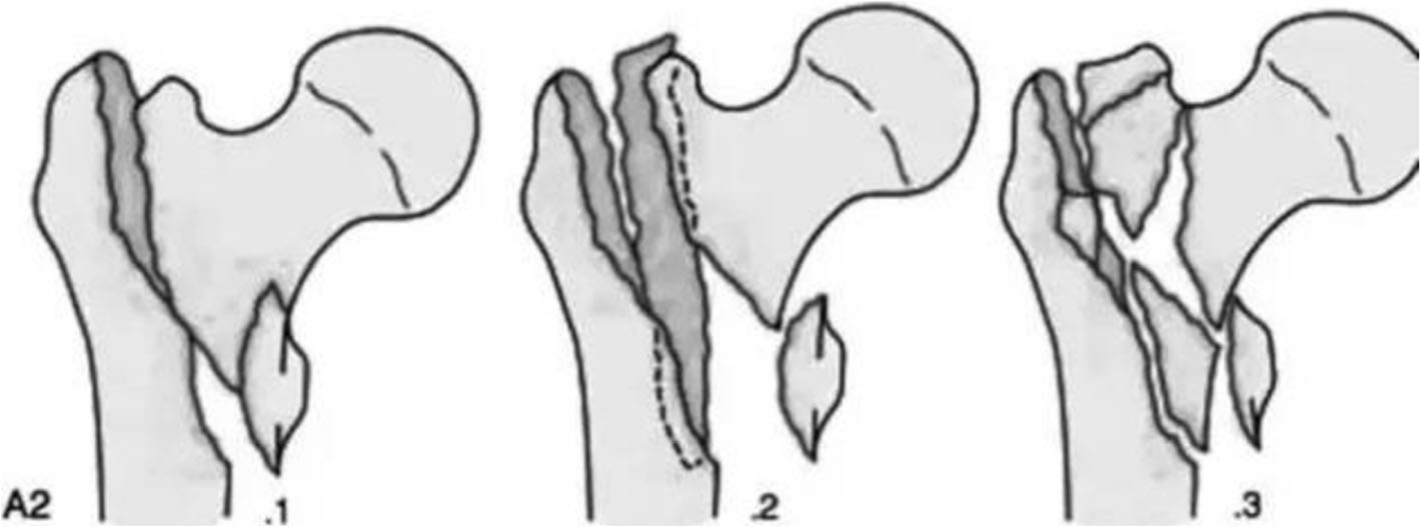
AO fracture classification. (A2) Transtrochanteric comminuted fracture, medial, and posterior bone cortex comminuted in more than two planes, lateral cortex intact. (A2.1) One medial fracture. (A2.2) More than two medial fracture blocks. (A2.3) The fracture line extending more than 1 cm under the lesser trochanter

**Table 2:** Results of the intraoperative indices (x ± s) for the three-point group and the routine group (x ± s) and the comparison results

Indicators	Three-point group	Conventional group	*T*	*P*
Total operation time (min)	59.34 ± 9.42	67.61 ± 12.63	3.560	< 0.01
Time to set the spiral blade (min)	4.58 ± 1.25	7.82 ± 2.19	8.671	< 0.01
Bleeding volume (ml)	92.78 ± 34.09	154.01 ± 39.10	7.922	< 0.01
Number of perspective for spiral blade	8.84 ± 1.45	14.62 ± 2.91	11.903	< 0.01
Apex distance (mm)	16.78 ± 1.55	21.91 ± 3.01	10.182	< 0.01

In the three-point group, there were 1 case of deep venous thrombosis, 2 cases of hypostatic pneumonia, and 1 case of urinary tract infection, which was cured after drug treatment; in the routine group, there were 2 cases of deep venous thrombosis and 3 cases of hypostatic pneumonia, which were cured after drug treatment. There was no significant difference in the incidence of complications between the two groups.

### Follow-up results

Eighty patients were followed up for an average of 12 months (10–15 months), including 42 cases in the three-point group and 38 cases in the routine group. During the follow-up period, there was no case of spiral blade incision in the three-point group, while 2 patients in the routine group had spiral blade *cutting out* from the femoral head 1 month after the operation. Six months after the operation, the Harris score was 83.17 ± 5.25 in the three-point group and 82.35 ± 4.72 in the routine group. There was no significant difference in Harris score between the two groups (*P* > 0.723).

## Discussion

In recent years, with the continuous development and improvement of internal fixation technology, PFNA has been widely used in the treatment of intertrochanteric fracture of the femur [7, 8]. It has the advantages of a short operation time, less trauma, a quick recovery, and a high fracture healing rate. The therapeutic effect has also been recognized by everyone [9, 10]. However, many X-rays and continuous adjustment are needed in the course of the operation to obtain a satisfactory therapeutic effect; especially in the process of inserting the femoral neck anti-rotating nail, many X-rays, continuous anteroposterior and lateral fluoroscopy, are required. The angle of the guiding needle can be adjusted repeatedly to achieve the ideal position [11, 12], but it is very difficult to adjust once the guiding needle is implanted; the operation time is prolonged, and there is a significant blood loss. Even so, the unreasonable screw cutout cannot be completely avoided. Previous studies have shown that poor reduction and incorrect spiral blade position of these fractures are associated with the occurrence of complications [13, 14]. The spiral blade has the problem of cutting and piercing out of the femoral head. With the continuous progress of technology, computer navigation technology has been gradually used in the process of operating, especially in operations for femoral neck fractures. Computer navigation can increase the accuracy of surgery, reduce trauma, reduce the radiation exposure of doctors and patients, protect patients and medical workers [15], and enable repeatability of the same operation [4]. However, due to the complexity of the equipment, it is only carried out in some large hospitals, requiring orthopedic surgeons to master the operating procedures. Over many years of performing operations for intertrochanteric fractures of the femur, the author’s team has found a simple and convenient method for inserting the spiral blade into the femoral neck (three-point positioning method), which adopts the principle of three points on one line. Before the operation, the author’s team accurately measured the neck shaft angle and the femoral neck anteversion, which was used to guide the reduction during the operation and to guide the inserting of the spiral blade. The three-point method is used to accurately locate the anteroposterior and lateral positions before and after the placement of the guiding needle, and the success rate is 100%, which greatly increases the accuracy of the operation; reduces the length of the operation, bleeding, and fluoroscopy time; and reduces the TAD. Compared with the common localization method, the difference was significant statistically. This method achieves the same results as navigation, such as avoiding excessive X-ray exposure and accurate placement of lag screws [16, 17].

In the process of intertrochanteric surgery, it is relatively easy to locate the apex of the greater trochanter and insert the main nail, and there is little deviation. The spiral blade needs to be placed in a middle or slightly lower position [18], but in the process of placement, we encounter all kinds of difficulties. First, in the AP position, the spiral blade needs to be hit in the center or slightly below the femoral neck and head, approximately 5 mm from the articular cartilage, and laterally on the central line between the femoral neck and the femoral head [19]. During the operation, anteroposterior fluoroscopy is needed first, the nail entry point at the greater trochanter is observed, the position under the femoral neck and femoral head that the guide needle should enter is observed, and the lateral fluoroscopy is changed to lateral fluoroscopy after satisfactory visual measurement. Because there is a forward inclination angle of approximately 15° in the femoral neck plane and the femoral shaft plane, it is necessary to observe the guide needle while looking at it. Once there is a large deviation in the angle, we need to withdraw the guide needle to adjust the angle and reinsert the needle. Moreover, if there is still a rear deviation of approximately 3° after adjustment due to the influence of multiple nail entry points and holes on the outside of the greater trochanter, it is very difficult to accurately adjust the guide needle in the center of the femoral neck, and cause helical blades were placed in different possible zones [20]. After many adjustments, when it was found that the lateral position is satisfactory and the positive perspective is viewed, it was found that it also deviates from the position where the original visual measurement was more satisfactory and needed to be adjusted again or that the less ideal position needed to be accepted, resulting in the inaccurate placement of the spiral blade. When increasing the TAD, there is an increase in the probability of the spiral blade cutting out of the femoral head and neck, which affects the effect of the operation and even leads to the failure of the operation [21]; we have encountered these problems in varying degrees with the use of regular fluoroscopy. This led to prolonged operation times, increased intraoperative bleeding, increased exposure times, and increased TAD. In the following up, we found two of patients with larger apex distances had screw cut, loss of reduction, and joint pain during walking. Finally, hip replacements were performed for the two patients.

The three-point positioning method is based on the principle that a straight line can be determined according to three points using geometry. Before placing the guiding needle, we began to accurately measure two angles and locate the marking point. Due to the existence of visual deviation, we first observed the center of femoral head and placed the guiding needle sleeve. First, the center of the sleeve, the femoral neck, and the center of the femoral head are measured with a ruler. When the central line of the sleeve overlaps with the central line of the femoral neck and the femoral head, visualization is changed to lateral fluoroscopy. Due to the existence of the anterior inclination angle, we use a tilt of approximately 15° to eliminate the forward inclination angle and reveal the pure lateral image of the femoral neck and then adjust the positioning frame before and after. When the center line of the sleeve, the center of the femoral neck, and the center of the femoral head overlap, the guiding needle is inserted directly. After all of this, the guiding needle was inserted directly, and the depth was approximately 5 mm below the joint surface. Using the three-point positioning method, the accurate needle placement rate was 100%, and the implantation time, bleeding volume, and fluoroscopic exposure times were significantly less than those of the common method. The average TAD was 16.78 mm, which was less than that of the common location method, and the difference was statistically significant.

Tip-apex distance (TAD) was proposed by Professor Baumgaertner in 1995. He believed that the TAD of the hip screw was less than 25 mm and could significantly reduce the failure rate of internal fixation [22]. Since then, many scholars have conducted research on the TAD and showed that a proper TAD can avoid implant failure [23, 24]. In 2004, Pervez et al. [25] conducted a retrospective study of 100 patients with hip fracture. The average TAD of the hip screw incision group was 27 mm, which was consistent with our experimental results. The results were not satisfactory. The TAD of two patients was more than 25 mm, while in the three-point positioning group, the TAD was less than 25 mm, and the average was 16.7 mm. None of the screws were cut out after the operation.

## Conclusions

In summary, the three-point positioning method is a set of methods determined by the author by his long-term clinical practice. This method only adopts the principles of geometry, adjusts the perspective angle, and locates the guiding needle in advance to achieve successful placement of anti-rotating nails, reduce intraoperative bleeding and fluoroscopy time, increase placement accuracy, and to decrease the cost of operation and the burden of patients; it can also be widely carried out in the clinic.

## Data Availability

Yes, data and material were available, not been published, and is not under consideration elsewhere.

## Abbreviations

PFNA: Proximal femoral nail anti-rotation
TAD: Tip-apex distance
